# In-vivo evaluation of analgesic and anti-inflammatory activities of the 80% methanol extract of *Acacia seyal* stem bark in rodent models

**DOI:** 10.1186/s40780-024-00387-1

**Published:** 2024-11-18

**Authors:** Gena Kedir, Akeberegn Gorems Ayele, Workineh Shibeshi

**Affiliations:** 1 Department of Pharmacy, College of Health Sciences, Mettu University, Mettu, Ethiopia; 2 Department of Pharmacology and Clinical Pharmacy, School of Pharmacy, College of Health Sciences, Addis Ababa University Addis Ababa, PO. Box: 1176, Emial, Ethiopia

**Keywords:** *Acacia Seyal*, Analgesic, Anti-inflammatory, Carrageenan, Pain

## Abstract

**Background:**

Pain and inflammation are the major medical condition commonly addressed with traditional remedies. *Acacia seyal* is a traditional herb widely used in Ethiopian folk medicine for pain management. However, its effectiveness has yet to be validated through scientific or experimental research. Therefore, the current study aims at evaluating the in vivo analgesic and anti-inflammatory effects of 80% methanolic stem bark extract of *Acacia seyal* in rodent models.

**Methods:**

After successful extractions of the stem barks of *Acacia seyal* with 80% methanol, the pain relieving effects of 100, 200 and 400 mg/kg extract were evaluated using acetic acid-induced writhing test and hot plate method whereas the anti-inflammatory profile was determined by carrageenan induced paw-edema model and cotton pellet induced granuloma technique.

**Results:**

The 80% methanol *Acacia seyal* stem bark extract exhibited substantial (*p* < 0.001) analgesic effect in acetic acid induced writing test (*p* < 0.001). The plant extract also witnessed significant central analgesic effect in hot plate method beginning at 30 min with maximum % elongation time occurred at 120 min. Furthermore, the acacia stem bark extract produced anti-inflammatory effect against carrageenan induced paw-edema model. In cotton pellet induced granuloma model, the 200 and 400 mg/kg doses of the current plant material appeared to inhibit granuloma mass formation and exudate reduction significantly (*p* < 0.001).

**Conclusion:**

The collective findings of the current study revealed that 80% methanol extracts of *Acacia seyal* exhibited considerable analgesic and anti-inflammatory activities, supporting the plant’s traditional use for management of pain and inflammatory disorders.

## Introduction

Pain and inflammation are the major medical condition commonly addressed with historical herbal remedies mostly by employing botanical medicines, mostly throughout the world, and they are common, nonspecific manifestations of many diseases [1]. These conditions are characterized by hallmark clinical manifestations of the cardinal sign like redness, swelling, and heat resulting from the involvement and releasement of substances like kinins, platelet-activating factor, prostaglandins (PGs), leukotrienes, amines, cytokines, and chemokines [2, 3]. The International Association for the Study of Pain defines pain as an unpleasant sensory and emotional experience associated with, actual or potential tissue damage [4, 5]. On the other hand inflammation, which is employed by both the innate and adaptive immune systems, is a protective response of the body to various noxious stimuli such as infections and tissue injury [6]. Although it is one of the body’s physiological mechanisms for maintaining homeostasis, effects of inflammation can sometimes extend beyond this balance. It can lower the pain threshold and lead to a state of hyperalgesia [7].

The management of pain dates back to ancient times, with the use of herbs like salicylate, originally prepared as decoctions by Greek and Roman clinicians [8]. Nowadays pharmacotherapy of pain relies on the use of a variety of medications, such as Non-steroidal anti-inflammatory drugs (NSAIDs) and opioid analgesics [9]. However, these drugs are associated with many side effects. Using NSAIDS for example results in gastric ulcer, gastric irritation, effects on blood pressure, alterations in renal function and hepatic injury, and platelet inhibition [10]. On the other hand, unwanted consequence such as dependence, sedation, constipation, and respiratory problem are common with opioids [11]. Medicinal plants have been a rich source of biologically active compounds, many of which hold significant potential for drug discovery and continue to serve as lead compounds in pharmaceutical development [12]. Thus, screening of medicinal plants known for their analgesic and anti-inflammatory properties may offer the opportunity to discover new compounds with enhanced safety and efficacy [13]. The experimental plant *Acacia seyal* (*A.seyal*) belongs to a family of Fabaceae and the huge genus Acacia, sometimes referred to as wattles [14] It is found abundantly in different parts of Ethiopia including Gojam, Shoa, Arsi, Harerge, Ilubabor, Kefa, Sidamo, western Tigray, and western Welo areas of Ethiopia [15].

Acacia has significant commercial and medical value. The majority of acacias have therapeutic benefits for local people. As an illustration, *Acacia nilotica* pods are used to cure toothaches, malaria, sore throats (aerial part), and wounds (pods) [16]. In different parts of African nations such as Burkina Faso, Niger, Kenya, Sudan, through decoctions of the stem bark, *A. seyal* has been used for the management of gastroenterology, hematology, and inflammatory disorders including Arthritis, rheumatisms, and rheumatoid fever. It is also employed as a pain relief in the case of joint and chest pain [17, 18]. Despite the traditional roles of *A.seyal* as analgesic and ant inflammatory, it lacks a scientific evidence. Thus, it is considered prudent to investigate these activities scientifically. Therefore, the aim of the present study is to evaluate the analgesic and anti-inflammatory activities of an 80% methanol stem bark extract of *A.seyal* in rodent models.

## Materials and methods

### Drugs and chemicals

The substances and pharmaceuticals used in this study include the following: distilled water (pharmaceutical department, AAU); indomethacin (Cadila pharmaceuticals, Ethiopia); carrageenan (Sigma Aldrich, Germany); ketamine (Neon laboratories, India); glacial acetic acid ((Lobe chemi, India); methanol (Cheshire, UK); morphine and acetyl salicylic acid (ASA) (Ethiopian Pharmaceuticals Manufacturing).

### Collection and identification the plant materials

The stem bark of *A. seyal* and leaves for identification have been collected from the Ziway Dugda district, located around 161 km away from Addis Ababa, in the Arsi zone of Oromia regional state. Identification and authentication of the plant specimens were conducted by a taxonomist at the National Herbarium, College of Natural and Computational Sciences, Addis Ababa University., and specimen with voucher number: GK001, was deposited for future reference.

### Animals used

An albino male Wistar rat (220–320 g) and Swiss albino mice of both sexes (20–30 g) aged 6–8 weeks were used to investigate the plant extract’s analgesic and acute anti-inflammatory activities. Some of the experimental animals were obtained from Ethiopina Public Health Institute (EPHI) and the rest was offered from the Addis Ababa University College of Health Science Department of Pharmacology and Clinical Pharmacy. The rodents were housed, and placed in polypropylene boxes, held at room temperature. They were given free access to water both before and throughout the experiment. The handling and euthanasia of animals were conducted in accordance with internationally recognized guidelines [19]. Additionally, the Institutional Review Board (IRB) of the School of Pharmacy approved all procedures.

## Methods

### Preparations of the experimental plant

After collecting, the stem barks of *A.seyal*, it was cleaned with running tap water to get rid of any dirt, dried for three weeks in the shade, and then pulverized into smaller size and macerated with 80% methanol for 72 h at room temperature. Thereafter, the extract was filtered through Whatman filter paper No. 1. After extraction, the methanol was allowed to be evaporated and concentrated using rotary evaporator and kept in a ventilated oven at 40 ºC until dried. Finally, the -dried extract was taken into a small glass tube and placed in the refrigerator until used.

### Acute oral toxicity study

Five female Swiss albino mice have been employed to evaluate the toxicity studies of the A. *seyal* in accordance with OECD Guideline 425 [20]. For this experiment, non-pregnant swiss albino female mice measuring weight 20–30 g and 8–12 weeks of age were chosen. A single female mouse, fasting for four hours was administered a dose of 2000 mg/kg of *A.seya* 80% methanol extract. Thereafter, the mice were watched for any changes in behavior or appearance, as well as any signs of death, with greater attention to first four hours. Following a 24-hour period, four additional mice were administered an equivalent dosage of the extract and monitored for a period of 14 days to assess general symptoms which included disturbances to the skin, eyes, behavior, and manifestations such as sweating, vomiting, loss of weight tremors, and mortality. The same approach was employed for evaluations of the toxicity of plant extract in rats. The outcomes of the acute oral toxicity study were taken into consideration when choosing three different doses of *A. seyal’s* stem bark extract.

### Animal grouping and dosing

Six animals per study group were randomly assigned to one of five categories (negative control, positive control, and three groups receiving 100 mg/kg (ASME100), 200 mg/kg (ASME200) and ASME400 mg/kg (ASME400) doses of *A. seyal* 80% methanol extract. In all models, the first group (negative control) received 10 ml/kg of distilled water whereas the second group (positive control) was given standard drug (20 mg/kg of morphine) for a hot plate method. Conversely, 150 mg/kg of aspirin for acetic acid induced writhing reflex, and indomethacin at 10 mg/kg was administered for positive control group in both carrageenan induced paw edema, and cotton pellet induced granuloma models.

## Analgesic activity test

### Acetic acid triggered writhing techniques

Based on grouping, after all animals administrated with their respective treatment, acetic acid was given intraperitoneally (10 mL/kg, i.p.) within an hour. After 5 min of latency, the plant extract’s pain reliving activity was measured by counting the numbers of writhing which consists of contraction of the abdominal muscle together with stretching of the hind limbs for 20 min. A reduction in the number of writhes as compared to the control group was considered as evidence for analgesic potential of the extract, and it was expressed as percent inhibition of writhing as follows [21] (Formula-1).

Formula-1 $$\begin{aligned}&\% \text{Analgesic activity} \cr&\quad = \frac{\text{(Mean no. writhes (control)}-\text{Mean no.f writhes (treated)})}{\text{(Mean no. writhes (control))}}\cr&\qquad\times 100\end{aligned}$$

### Hot plate method

In the hot plate method, the experimental animals were introduced into a metallic plate that would heat to a constant temperature of 55 °C +/- 1 °C. Paw biting, paw removal from the plate, and leaping or jumping out of plate were the main behavioral elements that were considered as a reaction time. After an overnight fast (12 h), animals were given the extract, vehicle or standard drug (morphine) as per grouping. Mice were individually placed on a hot plate with a cut-off time of 15 s to avoid damage to the animals’ paws [22]. The response time was measured by observing how long it took the mice to jump from the hot plate or lick their paws. Reaction times were measured to determine the percentage of elongation at 0, 30, 60, 90, and 120 min post administrations of each agent (Formula-2).

Formula-2 $$\%\: \text{Elongation} = \frac{\text{(latency (test)}-\text{latency(control))}}{\text{(latency (test))}} \times 100$$

## Anti-inflammatory activity test

### Paw edema induced by carrageenan

In this model, 1 h before injecting carrageenan (1% w/v in normal saline, 0.05mL) into the dorsal area of the right hind paw of mice, the standard drug, the vehicle and various doses of plant extract were given. Thereafter the thickness of the paw was determined with a micrometer at 0, 1, 2, 3, and 4 h following the carrageenan administration. Finally, the width of the swelling in the paw with respect to the mice that took distilled water was calculated using equations below [23] (Formula-3).

Formula-3 $$\% \:\text{Edema inhibition} = \frac{\text{(PEC-PET)}}{\text{PEC}} \times100$$

Where; PEC is paw swelling in control and PET is paw swelling in treatment group.

### Granulation developed by cotton pellet approach

Before the commencement of the experiment, all experimental animals (male rats weighing 220–350 g) were provided with free access to water and fasted for 12 h. Then, test animals were administered various doses of extract (100, 200 and 400 mg/kg) based on grouping whereas the positive group and negative control group received, indomethacin (20 ml/kg), and distilled water respectively. A cotton piece weighing 10 ± 1 mg was rolled into a pellet and autoclaved for 30 min at 120 degrees Celsius with 15 pounds of pressure to sterilize it. Rats were sedated with 1 ml/kg of ketamine, and 20 min following administrations of standard drug, extract or the vehicle, a subcutaneous tunnel was aseptically created using blunted forceps in the previously shaven groin area of each rat. Following their bilateral placement in the subcutaneous tunnel, the two were secured with a chromic catgut suture (0/4 metric, 1/2 circle). The standard agent and different doses of extract was administered (PO once daily) for 7 days in row, starting from the day that cotton pellets was placed in. After the rats had been killed under ketamine anesthesia on the 8th day, the pellets had been surgically removed and liberated from superfluous materials. The wet cotton was taken out and measured before being dried for 24 h at 60° C. The net dry weight was subsequently obtained by reducing the total mass of the cotton pellet from the total weight.

The following formulas were applied to compute the liquid exudate quantity (mg), the quantity of granulation tissue (mg), and the proportion of reduction in discharge and granulation development [24]. (Formula 4–5).

Formula-4 $$\text{Exudates reduction} (\%) = \frac{\text{(1-Exudates in treatment group)}}{\text{(Exudates in control group)}} \times 100$$

Formula-5 $$\text{Granuloma reduction} (\%) = \frac{\text{(1-Granuloma in Treatment group)}}{\text{(Exudates in control group)}} \times 100$$

Where,

Measure of Exudates = immediate wet weight of pellet − Constant dry weight of pellet,

Measure of granuloma **=** Constant dry weight of cotton − Initial weight of cotton pellet.

### Statistical analyses

All statistical analyses were performed using international business machine of statistical package for the social Sciences, (IBM SPSS), version 27 for windows (SPSS inc, Chicago, Illinois, USA). Statistical differences between groups was analyzed by one-way analysis of variance (ANOVA) followed by Tukey post hoc test. In all cases results were expressed as mean ± standard error of mean (SEM). P-values less than 0.05 were considered as statistically significant.

## Results

### Percentage yield

From 610 g of plant material, approximately 84.6 g of coarse powder was obtained, resulting in a 14% crude extract yield from the stem bark of *A. seyal*.

### Acute toxicity study

The outcomes of the acute toxicity test indicated that the plant extract was safe when orally given at a dosage of 2000 mg/kg in both animal species (rats and mice). Following 24 h, rodents (mice and rats) demonstrated tolerance to the given dose, with no notable alterations in behavior observed, including motor function, alertness, restlessness, diarrhea, or convulsions within the first 24 h post-administration. Additionally, no deaths were recorded during the 14-day observation period, suggesting that the median lethal dose of the extract (LD50) is ≥ 2000 mg/kg.

## Analgesic activity test

### Acetic acid induced writhing test

Acetic acid induced writing test was performed to evaluate the effects of extract in relieving peripheral pain induced by chemical stimuli. In contrast to the negative control group, all doses of the extract and the standard drug produced significant inhibition (*P* < 0.001) against acetic acid induced writhing compared to negative control (Table 1).

**Table 1:** Effect of 80% methanolic stem bark extracts of *A.seyal* against acetic acid induced writhing model in mice

Groups	Mean No. of Writhing ± SEM	Percentage Inhibition
DW	12.50 ± 1.03	
ASA150	5.50 ± 0.43 a^2^ b^2^ c^2^	56%
ASME100	10.50 ± 0.76a^2^	16%
ASME200	8.33 ± 0.42a^2^	33%
ASME400	6.50 ± 0.43 a^2^ b^2^	48%

Notes Each value represents mean ± S.E.M; *n* = 6 for each treatment; Analysis was performed by one way ANOVA; a, compared to negative control group; b, compared to ASME100; c, compared to ASME200;^2^*P*<0.001. Numbers followed by ASA and ASME indicate dose in mg/kg

Abbreviations: Abbreviation; DW, distilled water, ASME, *Acacia seyal* methanolic extract

When various doses of extract doses were compared, to each other, the highest dose of *A.seyal* methanol extract demonstrated a statistically significant (*p* < 0.001) decrease in the peripheral writhing response in contrast to the lower dose. Moreover, the group treated with the standard drug considerably lowers the frequency of writing compared to the ASME100 and ASME200. However, this change was not noticeable compared to the highest dose of the experimental plant.

### Hotplate test

When compared to the negative control at a 30 min, all doses of 80% methanol extracts of *A.seyal* exhibited considerable analgesic affect in hot plate method (*p* < 0.001) compared to negative control.

Although a significant latency period was not observed for ASME100 at time points other than 30 min, the effect of the middle dose of the plant extract persisted up to 120 min (*p* < 0.001), despite it failed to be noticed at 60 min. ASME400 produced appreciable latency period without interruption at any time points and its effect was noticed until the end of the study (120 min) (*P* < 0.001). Regarding the percentage of elongation, the greatest effect was observed at 120 min, with values of approximately 70%, 75%, and 76.62% for ASME100, ASME200, and ASME400, respectively (Table 2).

**Table 2:** Effects of 80% methanolic stem bark extracts of *A seyal* in hotplate test in Mice

Mean latency time (Sec) and percentage of elongation									
Groups	0 min	**30 min**		**60 min**		**90 min**		**120 min**	
		**Mean Latency**	**%**	**Mean Latency**	**%**	**Mean Latency**	**%**	**Mean Latency**	%
DW10	3.67 ± 0.21	2.67 ± 0.49		2 ± 0.25		2 ± 0.36		1.83 ± 0.31	
MRP10	2.67 ± 0.28	12.50 ± 0.75 a^2^b^2^c^2^	78.64	5.33 ± 0.61 a^1^	62.47	8.67 ± 1.38 a^2^	6.93	9.33 ± 0.49 a^2^	80.38
ASME100	5.33 ± 1.02	7.83 ± 0.60 a^2^	65.90	3.50 ± 0.67	42.85	4.67 ± 0.67	57.17	6.00 ± 0.68	69.50
ASME200	4.67 ± 0.71	8.17 ± 0.79 a^2^	67.32	4.83 ± 0.70	58.59	6.00 ± 0.97 a^1^	66.67	7.17 ± 0.47 a^2^	74.47
ASME400	4.50 ± 0.84	8.67 ± 0.61 a^2^	69.20	6.17 ± 1.35 a^1^	67.58	7.50 ± 0.76 b^2^	73.33	7.83 ± 1.18 a^2^	76.62

Notes Each value represents mean ± S.E.M; *n* = 6 for each treatment; Analysis was performed by one way ANOVA; a, compared to negative control group; b, compared to ASME100; c, compared to ASME200;^1^*P*<0.05;^2^*P*<0.001. Numbers followed by MRP and ASME indicate dose in mg/kg

Abbreviation: MRP, morphine; DW, distilled water; ASME, *A. seyal* methanolic extract

## Anti-inflammatory activity test

### Carrageenan induced paw edema

**Fig. 1:**
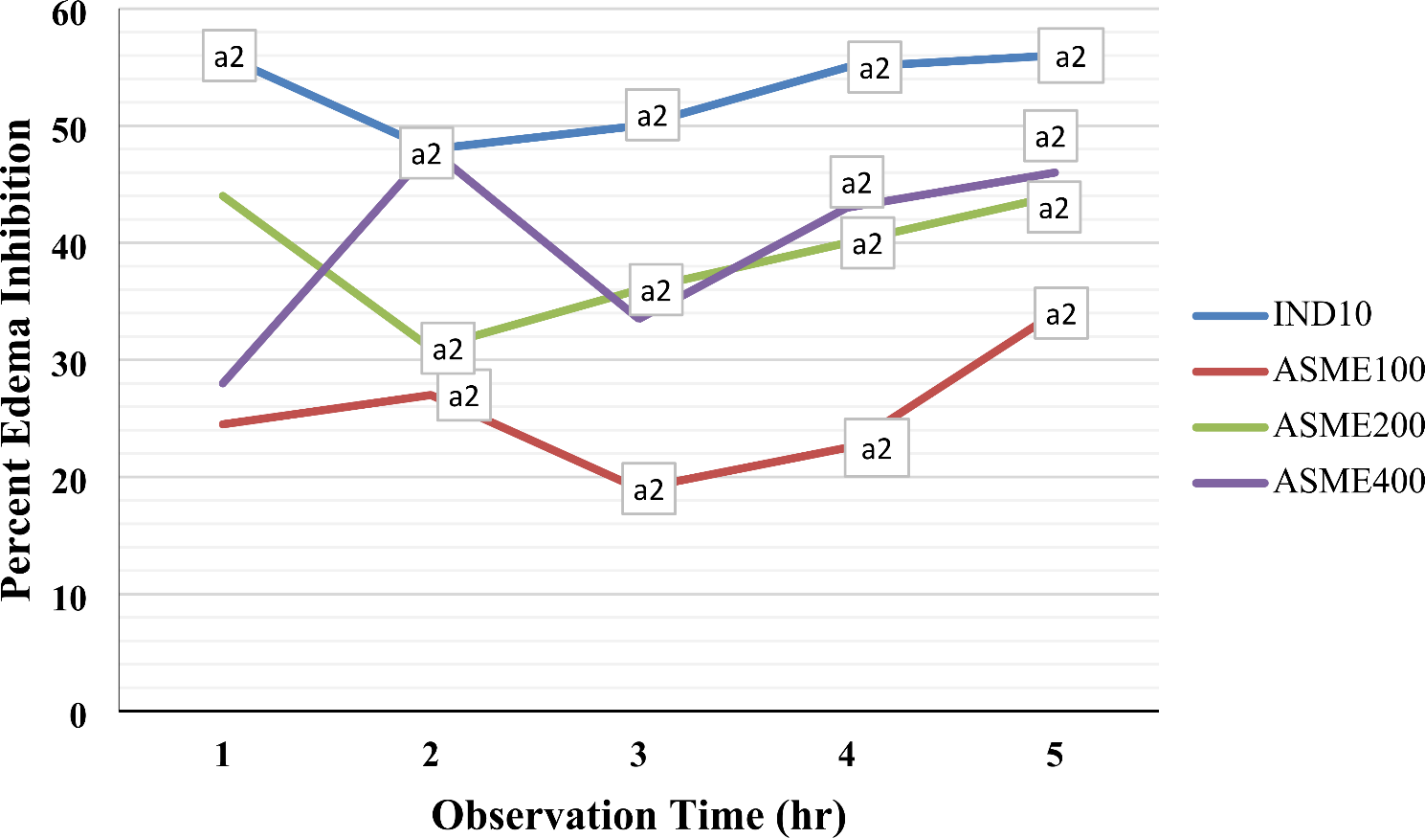
Percent edema inhibition of 80% methanol stem bark extract of Acacia seyal in carrageenan-induced paw edema in mice. Notes: Each value represents mean ± S.E.M; *n* = 6 for each treatment; Analysis was performed by one way ANOVA; a, compared to negative control group; Numbers followed by IND and ASME indicate dose in mg/kg. Abbreviation; IND, indomethacin; DW, distilled water; ASME, Acacia seyal methanolic extract

The carrageenan-caused edema model revealed that all doses of 80% the hydroalcoholic extracts of *A.seyal* produced significant inhibitory effects against carrageenan induced paw-edema beginning at 2 h and continued until the 5th hours of observation compared to negative control groups (*p* < 0.001) (Fig. 1). Likewise, the standard drug indomethacin witnessed significant inhibition 1 h earlier than the plant extract’s onset and this effect persists up unit the end of the experiment. The extract’s highest percentage of anti-inflammatory activity in across all extracts occurred at the 5th time point with the percentage of 34.5% for ASME100, 46.2% for ASME200, and 48.3% for the highest dose of extract.

### Cotton pellet induced granuloma method

In the cotton pellet induced granuloma, the medium and the maximum dose of 80% methanol extracts of *A.Seyal* appeared to reduce the development of granuloma mass formation significantly (*p* < 0.001)(Table 3). Furthermore, AMSE200 and ASME400 also produced an apparent reduction in the generations of the inflammatory exudate (*p* < 0.001) but the lowest dose plant extract failed to bring both granuloma mass formation and exudate reduction. In contrast to this, the standard drug exhibited significant effect in both parameters (*p* < 0.001).

Group analysis also revealed ASME400 demonstrates significant exudate and granuloma inhibition compared to ASME100 and ASME200.

**Table 3:** Anti-Inflammatory activity of 80% *A.seyal* on cotton pellet caused granuloma in rodents

Group	Exudate (Mean ± SEM)	% reduction of Exudation		Granuloma (Mean ± SEM)		% reduction of Granulation	
DW10	139.17 ± 4.14		38.91 ± 2.96				
IND10	92.74 ± 2.11^a2^b^2^d^1^	33.4%	14.37 ± 1.06a^2^b^2^c^1^		62.3%		
ASME100	125.49 ± 2.77	9.85%	32.73 ± 2.42		15.9%		
ASME200	112.448 ± 2.30a^2^	19.21%	26.35 ± 1.85a^2^b^1^		32.3%		
ASME400	104.51 ± 2.35a^2^b^2^	25%	20.59 ± 1.50a^2^b^1^		47.1%		

Notes Each value represents mean ± S.E.M; *n* = 6 for each treatment; Analysis was performed by one way ANOVA; a, compared to negative control group; b, compared to ASME100; c, compared to ASME200;^1^*P*<0.05;^2^*P*<0.001. Numbers followed by IND and ASME indicate dose in mg/kg

Abbreviation; IND, indomethacin; DW, distilled water; ASME, *Acacia seyal* methanolic extract

## Discussion

The current study evaluated the anti-inflammatory and pain relieving effects of *A. Seyal’s* 80% methanol stem bark extract [21]. Considering the socioeconomic significance of pain and inflammation together with the awareness of potential herbal medicines used for this condition, it would make reasonable to explore medicinal plant derived analgesic and anti-inflammatory agents as plant-derived agents are associated with minimal side effects and comparable efficacy [25]. *A. Seyal* is a traditional herb used to treat various inflammatory and pain disorders [26]. Nevertheless, to date, no scientific data is found concerning its pain relieving and anti-inflammatory properties in the literature. Therefore, to support its claimed traditional use, it could be worthwhile to do scientific research on the pain relieving and anti-inflammatory properties of *A. seyal* stem bark extract in rodent models.

In the first model, the analgesic effects of 80% methanol stem bark extract of *A. seyal* was investigated using acetic acid-induced writhing reflex model. Administrations of various doses of *A.seyal* extract considerably decreased the stomach twisting reactions following acetic acid administration when compared to the negative control group (*P* < 0.01). The observed inhibition activity was 16%, 33.36%, and 48% from ASME100, ASME200 and AMSE400 respectively, demonstrating a dose-dependent effect. The mechanism related to acetic acid triggered writhing reflex is mediated through increased production of PGs [27]. Furthermore, when acetic acid is injected through intraperitoneal site, it irritates the peritoneal cavity, which causes the release of natural inflammatory molecules such as histamine, serotonin, bradykinin and, substance P [21, 28]. This may indicate that the extract would elicit its action on peripherally situated pain transduction pathways through suppression of these inflammatory mediators.

The hotplate model was the second experimental technique employed to examine the pain relieving efficacy of *A. seyal* extract. This approach is a valuable tool for assessing centrally acting analgesic agent, that is widely used to enhances mice’s sensation of the pain in response to heat [29]. It is sensitive to strong analgesics and has a time limit of 15 s to minimize the duration rodents are exposed to the hot plate set at 55 ± 1 °C [30]. According to these models, the duration between the injection and the first behavioral response such as jumping or paw lickining is a measure of the analgesic action [31]. In the current experiment, administrations of the extract tend to produce analgesic effect beginning at 30 min with all doses (*p* < 0.001). The maximum effect was achieved at 2 h, as evidenced by percentage of elongation time of 69.5%, 74.47% and 76.62% for doses of 100, 200 and 400 mg/kg respectively. In consistence with this, positive control group which took morphine indicates significant anti-nociceptive at all interval of observation.

The third model, carrageenan-caused paw swelling, is another approach employed to evaluate the anti-inflammatory effects of substances [32]. Carrageenan is a potent molecule that imitates the release of pro and inflammatory mediators such as PGs, leukotrienes, histamine, and, bradykinin [33]. 1% carrageenan subplantar injections into the hind paw causes two stages of inflammation [34]. During one to two hour since administration, carrageenan cause bradykinin, 5-hydroxytryptamine, and histamine release, which are expected to be the reason for swelling. Throughout the second stage of edema formation, which lasted one to four hours, PG level is elevated [35]. The finding of the current investigation illustrates that ASME exhibited a statistically significant reduction of carrageenan induced paw edema (*p* < 0.001) across all time points except in the first hour compared to the negative control group. Similarly, the standard drug, indomethacin, significantly reduced paw edema starting from the first hour, with the effect lasting up to the 5th hour (*p* < 0.001).The noted swelling reduction was most significant in the last stage of inflammation, which mimics the possible effects of NSAID drugs like indomethacin. This implies that one of the possible anti-inflammatory effects of the current experimental plant might be due to inhibitions of the PG synthesis through blocking cyclooxygenase enzyme.

The cotton pallet-induced granulation approach is the final and fourth model. It is a popular techniques used for evaluating long-term anti-inflammatory potential of investigational agents [36]. The subcutaneous applications of a cotton pellet into a rodent results in the formation of a granuloma at the site of the implant. The initial events include accumulation of fluid and proteinaceous material together with an infiltration of macrophages, neutrophils, and fibroblasts, and multiplication of small blood vessels [13]. An increase in the moisture content of the cotton pellet indicates transudative phase of inflammation, whereas elevation in the dry density within the cotton pellet indicates the proliferative phase of inflammation [31]. The present study revealed that ASME100 (*P* < 0.05) and the middle and the highest doses of plant extract witnessed a significant reduction in the development of granuloma mass formation compared to the negative control group (*p* < 0.001). Moreover, middle and highest doses of the ASME reduces the production of inflammatory exudate significantly (*p* < 0.001) in contrast to DW administered group. The appreciable effect of the plant extract in this model might suggest that the extract could act through inhibiting transudative, exudative, and proliferative phases of subacute inflammation [36].

Following extraction using various solvents, findings have illustrate that stem and root bark of *A. seyal* contains flavonoids, saponins, terpenoids, steroids, alkaloids, phenols, and tannins [18]. Thus, the analgesic and anti-inflammatory effects of *A.seyal* is due to these secondary metabolites. For instance, flavonoids, tannins, and saponins are well known for their ability to inhibit pain perception and anti-inflammatory properties due to inhibition of enzymes involved in inflammation, especially synthesis of prostaglandins [37]. Furthermore, saponins, phenolic compounds, and terpenoids demonstrated profound anti-inflammatory and analgesic activity through reductions of the expressions of proinflammatory cytokines, particularly interleukin 1 and interleukin 6 [38, 39]. Studies have shown that the current plant material contains flavonoid compounds such as catechin and epicatechin, a triterpene isolated active constituent lupeol and a steroid compounds stigmasterol [26]. Catechin and epicatechin possess anti-inflammatory effect through free radical scavenging activity [40]. Furthermore, catechins can exert their significant anti-inflammatory properties by regulating the activation or deactivation of inflammation-related oxidative stress-related cell signaling pathways, such as mitogen activated protein kinases, nuclear factor-kappa B and transcription factor nuclear factor (erythroid-derived 2)-like 2 [41]. On the other hand, lupeol’s analgesic and anti-inflammatory effect is mediated through inhibitions of Inducible nitric oxide synthase thus the pro-inflammatory productions of Nitric oxide [42] and steroid compounds including stigmasterol abolish inflammation through decreasing leukocyte infiltration [43].

## Conclusion

The collective results of our study unequivocally demonstrated the strong anti-inflammatory and analgesic activity of the 80% *A.seyal* plant extract, lending empirical support to its traditional claim as a natural remedy for inflammatory and painful conditions. The findings may indicate that the plant extract was involved in blocking the activity of several physiological mediators of inflammation, molecules involved in pain pathways, and regulations and this effect might be due to the presence of phytochemical constituents including flavonoids, saponins, terpenoids, steroids, alkaloids, phenols, and tannins. The current findings put scientific evidence about the traditional claimed uses of *A.seyal* for inflammation and painful conditions in Ethiopian folk medicines.

## Data Availability

The datasets used in this study are available from the corresponding author upon request.

## Abbreviations

ASA: Acetyl Salicylic Acid
DW: Distilled Water
NSAIDs: Non-steroidal Anti-Inflammatory Drugs
OECD: Organization for Economic Co-operation and Development
PGs: Prostaglandins

## References

[1] Tatiya AU, et al. Evaluation of analgesic and anti-inflammatory activity of Bridelia retusa (Spreng) bark. J Traditional Complement Med. 2017;7(4):441–51. 10.1016/j.jtcme.2016.12.009 PMC5634739 29034192

[2] Elgorashi E, McGaw L. African plants with in vitro anti-inflammatory activities: a review. South Afr J Bot. 2019;126:142–69.

[3] Sourabie T, et al. Biological evaluation of anti-inflammatory and analgesic activities of Argemone mexicana Linn.(Papaveraceae) aqueous leaf extract. Int J Pharm Sci Res. 2012;3(9):451–8.

[4] Alemu A et al. Analgesic and anti-inflammatory effects of 80% methanol extract of Leonotis ocymifolia (burm. F.) iwarsson leaves in rodent models. Evidence-Based Complementary and Alternative Medicine, 2018. 2018. 10.1155/2018/1614793 PMC5838498 29675050

[5] Deuis JR, Dvorakova LS, Vetter I. Methods used to evaluate pain behaviors in rodents. Front Mol Neurosci. 2017;10:284. 28932184 10.3389/fnmol.2017.00284 PMC5592204

[6] Organization WH. WHO guidelines on the pharmacological treatment of persisting pain in children with medical illness. No Title); 2012. 23720867

[7] Murray CB, et al. The prevalence of chronic pain in young adults: a systematic review and meta-analysis. Pain. 2022;163(9):e972–84. 34817439 10.1097/j.pain.0000000000002541

[8] Steinmeyer J. Pharmacological basis for the therapy of pain and inflammation with nonsteroidal anti-inflammatory drugs. Arthritis Res Therapy. 2000;2(5):1–7. 10.1186/ar116 PMC130140 11094452

[9] Sardana V, et al. Are non-steroidal anti-inflammatory drug injections an alternative to steroid injections for musculoskeletal pain? A systematic review. J Orthop. 2018;15(3):812–6. 10.1016/j.jor.2018.08.022 PMC6104144 30140124

[10] Geremew H, et al. Experimental evaluation of analgesic and anti-inflammatory activity of 80% methanolic leaf extract of Moringa Stenopetala Bak. F. in mice. Ethiop Pharm J. 2015;31(1):15.

[11] Baldini A, Von Korff M, Lin EH. A review of potential adverse effects of long-term opioid therapy: a practitioner’s guide. Volume 14. The primary care companion for CNS disorders; 2012. p. 27252. 3. 10.4088/PCC.11m01326 PMC3466038 23106029

[12] Ravalji M, Cevallos-Arellano E, Balakrishnan S. Investigation of centrally and peripherally acting analgesic and anti inflammatory activity of biological immune response modulator (an amazonian plant extract) in animal models of pain and inflammation. Int J Basic Clin Pharmacol. 2015;4:342–8.

[13] Patil KR, et al. Animal models of inflammation for screening of anti-inflammatory drugs: implications for the discovery and development of phytopharmaceuticals. Int J Mol Sci. 2019;20(18):4367. 31491986 10.3390/ijms20184367 PMC6770891

[14] Negash L. A Selection of African native trees: Biology, uses, propagation and restoration techniques. Addis Ababa, Ethiopia; 2021.

[15] Mengesha AK, Birru EM, Adugna M. Anti-diarrheal activities of hydromethanolic crude extract and solvent fractions of acacia seyal (Fabaceae) roots in mice. Clin Pharmacol Adv Appl. 2022;14:99–110. 10.2147/CPAA.S383896 PMC9675325 36411816

[16] Jacknoon AA, et al. A preliminary qualitative study of two common Acacia species in Sudan. J Chem. 2012;9:851–6.

[17] Subhan N, et al. Phytochemistry, ethnomedicine, and pharmacology of Acacia. Stud Nat Prod Chem. 2018;57:247–326.

[18] Magnini R, et al. A review on ethnobotanical uses, biological activities and phytochemical aspects of Acacia senegal (L.) Willd. And Acacia seyal Delile.(Fabaceae). Int J Plant Sci Hortic. 2020;2:32–55.

[19] 안나. Studies on the Institutional Animal Care and Use Committee operation strategy in Korea. 2022, 서울대학교 대학.

[20] OECD. Guideline fortesting of Chemicals, Guideline 423: acute Oral Toxicity-Acute Toxic Class Method 2001. 2001.

[21] Yimer T et al. Evaluation of analgesic and anti-inflammatory activities of 80% methanol root extract of Echinops Kebericho M. (Asteraceae). J Inflamm Res. 2020;13:647–58. 10.2147/JIR.S267154 PMC7533268 33061529

[22] Ashenafi E et al. Analgesic and anti-inflammatory effects of 80% methanol extract and solvent fractions of the leaves of Vernonia Auriculifera Hiern. (Asteraceae). J Exp Pharmacol. 2023;15:29–40. 10.2147/JEP.S398487 PMC9888398 36733956

[23] Marzouk B, et al. Screening of analgesic and anti-inflammatory activities of Citrullus colocynthis from southern Tunisia. J Ethnopharmacol. 2010;128(1):15–9. 19962436 10.1016/j.jep.2009.11.027

[24] Aziz TA, et al. Anti-inflammatory activity of silibinin in animal models of chronic inflammation. Am J Pharmacol Sci. 2014;2(1):7–11.

[25] Tesfaye R et al. Evaluation of analgesic and anti-inflammatory potential of 80% methanol leaf extract of otostegia integrifolia benth (Lamiaceae). J Inflamm Res. 2020;13:1175–83. 10.2147/JIR.S285932 PMC7767723 33380820

[26] Ashour MA, et al. A review on the main phytoconstituents, traditional uses, inventions, and patent literature of gum arabic emphasizing Acacia seyal. Molecules. 2022;27(4):1171. 35208961 10.3390/molecules27041171 PMC8874428

[27] Barba-Ostria C, et al. Evaluation of biological activity of natural compounds: current trends and methods. Molecules. 2022;27(14):4490. 35889361 10.3390/molecules27144490 PMC9324072

[28] Demsie DG et al. Anti-nociceptive and anti-inflammatory activities of crude root extract and solvent fractions of Cucumis ficifolius in mice model. J Pain Res. 2019;12:1399–409. 10.2147/JPR.S193029 PMC6504711 31118758

[29] Fan S-H, Ali NA, Basri DF. Evaluation of analgesic activity of the methanol extract from the galls of Quercus infectoria (Olivier) in rats. Evidence-Based Complementary and Alternative Medicine, 2014. 2014.

[30] Modi AD, Parekh A, Pancholi YN. Evaluating pain behaviours: widely used mechanical and thermal methods in rodents. Behav Brain Res. 2023;446:114417. 37003494 10.1016/j.bbr.2023.114417

[31] Ashagrie G, Abebe A, Umer S. Analgesic and anti-inflammatory activities of 80% methanol extract and solvent fractions of ehretia cymosa thonn (boraginaceae) leaves in rodents. J Exp Pharmacol. 2023;15:63–79. 10.2147/JEP.S396769 PMC9970881 36864852

[32] Khedir SB et al. Research article in vivo evaluation of the anti-inflammatory effect of Pistacia lentiscus Fruit Oil and its effects on oxidative stress. 2016. 10.1155/2016/6108203 PMC5192325 28070202

[33] Amdekar S et al. Anti-inflammatory activity of lactobacillus on carrageenan-induced paw edema in male wistar rats. International journal of inflammation, 2012. 2012. 10.1155/2012/752015 PMC3299308 22518342

[34] Radhakrishnan R, Moore SA, Sluka KA. Unilateral carrageenan injection into muscle or joint induces chronic bilateral hyperalgesia in rats. Pain. 2003;104(3):567–77. 12927629 10.1016/s0304-3959(03)00114-3 PMC2732018

[35] Zhao J et al. Evaluation on analgesic and anti-inflammatory activities of total flavonoids from Juniperus sabina. Evidence-Based Complementary and Alternative Medicine, 2018. 2018. 10.1155/2018/7965306 PMC6057303 30069226

[36] Dzoyem J, et al. Anti-inflammatory and anti-nociceptive activities of African medicinal spices and vegetables. Medicinal spices and vegetables from Africa. Elsevier; 2017. pp. 239–70.

[37] Serafini M, Peluso I, Raguzzini A. Flavonoids as anti-inflammatory agents. Proc Nutr Soc. 2010;69(3):273–8. 20569521 10.1017/S002966511000162X

[38] Turner R. Screening methods in pharmacology. Elsevier; 2013.

[39] Sulaiman MR, et al. Antinociceptive activity of the essential oil of Zingiber zerumbet. Planta Med. 2010;76(02):107–12. 19637111 10.1055/s-0029-1185950

[40] Bae J, et al. Activity of catechins and their applications. Biomedical Dermatology. 2020;4:1–10.

[41] Fan FeiYan FF, Sang SL, LiXuan. and J.M. Jiang Min, Catechins and their therapeutic benefits to inflammatory bowel disease. 2017. 10.3390/molecules22030484 PMC6155401 28335502

[42] Schmid D, et al. Inhibition of inducible nitric oxide synthesis by Cimicifuga racemosa (Actaea racemosa, black cohosh) extracts in LPS-stimulated RAW 264.7 macrophages. J Pharm Pharmacol. 2009;61(8):1089–96. 19703353 10.1211/jpp/61.08.0013

[43] Morgan LV, et al. Investigation of the anti-inflammatory effects of stigmasterol in mice: insight into its mechanism of action. Behav Pharmacol. 2021;32(8):640–51. 34657071 10.1097/FBP.0000000000000658

